# Analysis toxicity by different methods and anxiolytic effect of the aqueous extract *Lippia sidoides* Cham.

**DOI:** 10.1038/s41598-022-23999-9

**Published:** 2022-11-30

**Authors:** Cicera J. Camilo, Débora O. D. Leite, Johnatan W. da S. Mendes, Alexandro R. Dantas, Natália K. G. de Carvalho, José W. G. Castro, Gerson J. T. Salazar, Maria Kueirislene Amâncio Ferreira, Jane Eire Alencar de Meneses, Antonio Wlisses da Silva, Helcio S. dos Santos, Josean F. Tavares, Joanda P. R. e Silva, Fabiola F. G. Rodrigues, Chunhoo Cheon, Bonglee Kim, José Galberto Martins da Costa

**Affiliations:** 1 Postgraduate Program in Ethnobiology and Nature Conservation, Federal Rural University of Pernambuco, R. Dr. Miguel, Parnamirim, PE 56163-000 Brazil; 2 Northeast Biotechnology Network-RENORBIO, Graduate Program in Biotechnology, State University of Ceará, Fortaleza, Ceará 60714-903 Brazil; 3 Postgraduate Program in Biological Chemistry, Department of Biological Chemistry, Regional University of Cariri, Crato, Ceará 63105-00 Brazil; 4 Natural Products Research Laboratory, Regional University of Cariri, Crato, Ceará 63105-00 Brazil; 5 Graduate Program in Biological Diversity and Natural Resources, Regional University of Cariri, Crato, Brazil; 6 Multiuser Laboratory of Characterization and Analysis, Federal University of Paraíba, João Pessoa, 58051-900 Brazil; 7 Korean Medicine-Based Drug Repositioning Cancer Research Center, College of Korean Medicine, Kyung Hee University, Kyungheedae-Ro 26 Dongdaemun-Gu, Seoul, 05254 South Korea; 8 Postgraduate Program in Natural Sciences-PPGCN, State University of Cear, Fortaleza, Ceará Brazil

**Keywords:** Biological techniques, Biotechnology, Chemical biology, Ecology, Plant sciences

## Abstract

*Lippia sidoides* Cham. (Verbenaceae) is a species often mentioned in traditional medicine due to the medicinal properties attributed to its leaves, which include antibacterial, antifungal, acaricidal and antioxidant. Several of these actions have been scientifically proven, according to reports in the literature; however, little is known about toxicological aspects of this plant. This work included studies to determine the chemical composition and toxicity tests, using several methods aiming to evaluate the safety for use of the aqueous extract of *L. sidoides* leaves, in addition, the anxiolytic effect on adult zebrafish was investigated, thus contributing to the pharmacological knowledge and traditional medicine concerning the specie under study. The chemical profile was determined by liquid chromatography coupled to mass spectrometry-HPLC/MS with electrospray ionization. Toxicity was evaluated by zebrafish, *Drosophila melanogaster*, blood cells, and *Artemia salina* models. 12 compounds belonging to the flavonoid class were identified. In the toxicity assays, the observed results showed low toxicity of the aqueous extract in all tests performed. In the analysis with zebrafish, the highest doses of the extract were anxiolytic, neuromodulating the GABAa receptor. The obtained results support the safe use of the aqueous extract of *L. sidoides* leaves for the development of new drugs and for the use by populations in traditional medicine.

## Introduction

Natural products of vegetal origin are recognized for the variety of chemical substances present in their parts, and for their broad property of performing pharmacological activities^1^. Although the industrial influence, medical plants are still used to cure and treat different diseases by the majority of population^2^. The medical use can occur through different ways of preparations, whether in the form of teas, stews, baths, among others^3^. These plants represent considerable importance for many different cultural groups, since they are accessible and can be used against several types of pests and diseases^4^.

In this regard, recognizing the potential of a particular plant species to present some type of toxic reaction among its users is important, whether for the development of new drugs or for home use based on preparations; consequently, an evaluation of the relationship between risk and benefit is necessary to improve medical plant use, which means that the development of complete toxicological studies to help ensure the use of these species is extremely important.

The search for anxiolytic drugs has become relevant in current research, given that the most used drug in the clinic is Diazepam^5^, but clinical uses of benzodiazepines are limited by their side effects such as psychomotor impairment, sedation, myorelaxation, ataxia, amnesia, physical and psychological dependence^6^. Benzodiazepines act through benzodiazepine receptors present on the pentameric GABAa complex.

The genus *Lippia*, related to the family Verbenaceae, contains about 200 species distributed in various parts of the world. The species Lippia sidoides, popularly known as “Alecrim-pimenta” (rosemary pepper), is an aromatic plant which can be found in the Northeast region of Brazil^7^. In traditional medicine, this species is used to treat inflammation, wounds, mycoses, and acnes^8^; it is also part of the list of Brazilian medical plants, which are considered of interest by RENISUS (The National List of Medicinal Plants of Interest to the Brazilian Unified Health System (SUS)).

Many studies have demonstrated the pharmacological potential of products extracted from *L. sidoides*, such as antimicrobial activity^9,10^, acaricidal^11^, anti-inflammatory, antioxidant, and gastroprotective^12^; these activities are generally associated with the essential oil, with some chemical components highlighted: E-caryophyllene, thymol, 1,8-cineol, β-myrcene, and other important substances.

*Lippia* essential oils are associated with different biological activities, such as antimicrobial. In randomized studies have verified the effect of mouthwash based on *L. sidoides* essential oil to reduce dental plaque, gingival inflammation and gingival bleeding. The random choice of patients made it possible to verify the efficiency of the oil treatment over the course of seven days. In one of the studies, a decrease in the presence of Streptococcus mutans among treated patients is demonstrated^13,14^. This information refers to the fact that there is little research demonstrating the biological capacity of its fixed extracts, especially related to toxicity^15,16^.

The aqueous extract of *L. sidoides* leaves has compounds such as quercetin, luteolin and taxifolin, which are responsible for important pharmacological activities. Among these activities, anxiolytic activity was recorded by quercetin and luteolin, which may have their effect due to interactions with GABAergic receptors^17,18^.

Knowing the pharmacological potential that the species *L. sidoides* presents and its variability of chemical compounds considered active, it is important to develop new studies that can consider this species as a future phytotherapic.

Considering the importance of its extracts for the development of scientific studies, and the necessity to assure the use of this species by the population in traditional medicine, the present study approaches the toxicological profile of the aqueous extract of *Lippia sidoides* leaves by different evaluation methods and its anxiolytic effect on adult zebrafish via GABAa receptor.

## Materials and methods

### Obtaining plant material and preparing the extract

Experimental research and field study, including collection of plant material, followed institutional, national and international guidelines and legislation. Permission to collect leaves of *Lippia sidoides* was obtained from the authority of the Regional University of Cariri and the registration is in the Genetic Heritage Management Council of Brazil (code A2B7A05) and in the Herbário Caririense Dárdano de Andrade Lima, from URCA, under number 3038.

*Lippia sidoides* leaves were collected from the medicinal plant garden of the Universidade Regional do Cariri-URCA; latitudinal coordinates 7° 14′ 20.1″ S, and longitudinal coordinates 39° 24′ 53.1 W. The collecting was performed on November 2019 at 3 h in the afternoon.

Fresh leaves were macerated with hexane to remove lipidic components and then subjected to agitation with water in a refrigerated Shaker incubator (NT 715 Novatecnica) using the following parameters: Temperature: 50 °C; Rotation: 180 rpm; Agitation time: 4 h/day for 7 days. The yield corresponded to 4 L of aqueous solution, which was dried by atomization until obtaining the powder form with the Mini-spray dryer MSDi 1. 0 Mini-spray dryer (Labmaq do Brasil), using a 1.2 mm nozzle, under the following operational conditions: (a) flow control: 200 mL/h; (b) inlet temperature: 120 ± 2 °C; (c) outlet temperature: 88 ± 2 °C; (d) atomization air flow: 45 L/min; (e) blower flow: 1.80 m^3^/min.

### HPLC–MS-ESI analysis

The extract was analyzed by HPLC system—Shimadzu, using analytical chromatographic column C18 (Kromasil—250 mm × 4.6 mm × 5 μm), coupled to a mass spectrometer (Ion-Trap AmazonX, Bruker) with Electrospray Ionization (ESI). The sample was solubilized in methanol (1 mg/mL), after which it was filtered on PVDF (Poly-vinylidene Fluoride) filters with a mesh size of 0.45 μm. The chromatographic method used methanol (solvent B) chromatographic grade and ultrapure water type I (Milli-Q) with formic acid (0.1% v/v) (solvent A), in concentration gradient (5–100% of B in 95 min). The injection volume was 10 μL and flow rate was 0.6 mL/min. In the mass spectrometer, the samples were subjected to sequential fragmentation in MS3. The parameters used were: capillary 4.5 kV, end plate offset 500 V, nebulizer gas 35 psi, dry gas (N2) with flow rate 8 mL/min and temperature 300 °C. The sample was analyzed in negative ionization mode and the identification of the compounds was based on the data (MS/MS) as reported in the literature.

### Total flavonoid quantification

The quantification of flavonoids was performed according to the methodology described by Kosalec et al.^19^ with adaptations. The extract was prepared at an initial concentration of 20 μg/mL and diluted by 10; 5; 2 and 1 μg/mL in tubes with a final volume of 50 mL. Into these tubes, a total of 760 μL of methanol, 40 μL of 10% potassium acetate, 40 μL of 10% aluminum chloride, and 1,120 mL of water were added; the samples were incubated at ambient temperature, and readings taken in a UV–visible spectrophotometer at 415 nm. The analysis was performed in triplicate, and the flavonoid content calculated from the calibration curve using quercetin (QE), with results expressed as µg eq Q/g for extract.

### Toxicity analysis in *Drosophila melanogaster*

#### Rearing and stocking of *Drosophila melanogaster*

*Drosophila melanogaster* (Harwich strain) was obtained from the National Species Stock Center, Bowling Green, OH. Flies were reared in 340 mL glass containers with the medium containing: (83% corn paste, 4% sugar, 4% freeze-dried milk, 4% soybean meal, 4% wheat bran, and 1% salt). When cooking the mixture, 1 g Nipagin (Methylparaben) was added. After cooling in the growth containers, 1 mL of solution containing Saccharomyces cerevisiae was added. The flies were kept at 25 °C and 60% relative humidity. All tests were performed with the same strain^20^.

### Mortality test

Tests were performed according to the method proposed by Cunha et al.^20^ with some modifications. Adult flies (males and females) were placed in 130 mL glass containers (6 cm high and 6.5 cm in diameter), containing filter paper at the bottom. For the control, 1 mL of 20% sucrose in distilled water was added on this paper. For the other groups different concentrations of the extract ranging from 2000 to 4000 µg/mL were added. During the entire procedure, a 12-h light/dark cycle was maintained with a controlled temperature of 25 °C, and 60% relative humidity. The experiment was performed in triplicate in which each "n" was composed of two containers, with 20 flies placed in each of them. Readings to verify mortality were taken every 3, 6, 12, 24 and 48 h.

### Negative Geotaxis Test

Determination of the damage to locomotor ability was performed using the negative geotaxis assay. Each group of live flies exposed to the different concentrations of the extract at reading times of 3, 6, 12, 24 and 48 h were led to the bottom of the containers, and after 1 min the number of flies that reached 8 cm in height from the container were counted. The trials were repeated twice at 1 min intervals^21^.

### Cytotoxic activity on erythrocytes

#### Preparation of human erythrocytes and ethics statement

The erythrocyte samples were donated from the blood bank of the Laboratory Escola de Análises Clínicas de Biomedicina, with the consent and approval of the responsible researcher, José Walber Gonçalves Castro-CRBM 9815. The developed method was reviewed and approved by the biomedicine clinical analysis committee from the Doctor Leão Sampaio University Center. The procedures were performed according to the laboratory immunohematology manual-Ministry of Health, Brazil.

The blood used was type O^+^, which was initially homogenized in sodium citrate before the procedure. In a test tube, 900 µL of saline solution was pipetted, followed by 100 µL of whole blood in sodium citrate. The red blood cell washing procedure was performed by centrifuging the tube at 3500 rpm for 15 s, discarding the supernatant at the end of each centrifugation. The washing process was repeated 6 times, removing as much of the supernatant as possible with absorbent paper on the last wash. At the end the RBCs were homogenized with 900 µL of saline.

### Cytotoxicity analysis

Blood samples were collected, prepared, and exposed to different concentrations of the extract (10, 25, 50, 100, 250, 500, 1000 µg/mL). The solutions remained in a 37 °C water bath for 30 min, after which 2100µL of 0.9% saline solution was added to the blood. Following this, the samples were centrifuged, and the supernatant was read in a UV–visible spectrophotometer at a wavelength of 540 nm. The negative control contained only the RBCs and 0.9% saline solution^22^.

### Morphological analysis

The samples treated with the aqueous extract of *L. sidoides* and the positive control were prepared and fixed on slides for microscopic analysis. The images were obtained by smears of erythrocytes stained by conventional staining and compared using specific software for erythrocyte morphological analysis based on morphological changes described in the literature.

### Cytotoxic activity against *Artemia salina*

*Artemia salina* eggs were incubated in artificial seawater under light at 28 °C. After 24 h of incubation, larvae were collected with a Pasteur pipette, and kept for another 24 h under the same conditions to reach the largest stage. The sample was dissolved in Tween 80 and serially diluted (1000, 250, 125, 100, 75 μg/mL) in seawater; Next, 10 larvae were added to each set of tubes containing the samples. A control was run with potassium dichromate. 24 h later, the number of survivors was counted^23^.

### Zebrafish bioassays

#### Zebrafish

Adult zebrafish (ZFa) animals between 60 and 90 days (0.4 ± 0.1 g), from the wild, of both sexes, were obtained from a commercial supplier (Fortaleza, CE). The animals were kept in glass aquarium (n = 5/L), at a temperature of 25 ± 2 °C, in light–dark cycles for 24 h. Water was treated with antichlorine. The bioassays performed are in accordance with the Ethical Principles of Animal Experimentation, and were approved by the Ethics Committee for Animal Use (CEUA) of the Ceará State University (UECE) (04983945/2021). The procedures for performing this test are in accordance with the ARRIVE guide. After the experiments, the animals were sacrificed by freezing and immersed in ice water (2–4 °C) for 10 min until loss of opercular movements.

### Locomotor activity (Open Field Test)

Animals were given the sample application, and subsequently submitted to the open field test^24^ to assess whether there was a change in the motor system, either by sedation and/or muscle relaxation. Animals (n = 6/group) were intraperitoneally treated with the extracts (40; 200 and 400 mg/kg; 20 µL; i.p), and group with vehicle (DMSO 3%). One group of animals (n = 6/group) without treatments (Naive) was included. After 30 min of the treatments, the animals were added into Petri dishes (10 × 15 cm) containing the same aquarium water, marked with four quadrants, and analyzed locomotor activity by counting the number of line crossings (CL). Using the CL value of the Naive group as a baseline (100%), the percentage of line crossings (CL%) was calculated individually for 0–5 min.

### Acute toxicity 96 h

The acute toxicity study was conducted against adult zebrafish according to the Organization for Economic Cooperation and Development Standard Method^25^ to determine the LD50-96 h. Mortality was controlled every 12 h after the beginning of the tests. The animals (n = 6/group) were treated intraperitoneally with 20 µL of the extracts (40; 200 and 400 mg/kg; 20 µL; i.p), vehicle (DMSO 3%). After 96 h, number of fish deaths in each group were counted and the lethal dose capable of killing 50% of the animals (LD_50_) was determined using the Trimmed Spearman-Karber method with 95% confidence interval^26^.

### Anxiolytic activity (light–dark test)

The animals’ anxiety behavior was observed using a light–dark test. Similar to rodents, adult zebrafish naturally avoid illuminated areas^27^. The experiment was carried out in a glass aquarium (30 cm × 15 cm × 20 cm) divided into light and dark areas. The aquarium was filled with non chlorine tap water, which simulated a new shallow environment different from the conventional aquarium and capable of inducing anxiety behaviors. In animals (n = 6/group), 20 μL of the extract was administered intraperitoneally (i.p.) (40; 200 and 400 mg/kg; 20 µL). Negative and positive control groups consisted of 3% DMSO and 10 mg/kg DZP solution, respectively. After 1 h, the animals were placed individually in the clear zone, and the anxiolytic effect was measured based on the time spent in the clear zone of the aquarium within 5 min of observation^28^.

### Assessment of GABAergic neuromodulation

The anxiolytic action mechanisms of the extract were identified through pretreatment with flumazenil (a benzodiazepine channel antagonist)^29^. Zebrafish (n = 6/group) were pretreated with flumazenil (4 mg/kg; 20 μL; i.p.). After 15 min, the highest effective dose of the extract with an anxiolytic effect (400 mg/kg; 20 μL; i.p.) found in the pilot test was administered (see previous section). A group treated with 3% DMSO (vehicle; 20 μL; i.p.) was used as a negative control. DZP (10 mg/kg, 20 μL; i.p) was used as a positive control because it is an agonist of the Benzodiazepine binding channel in GABAa. After 30 min of treatment, the animals were submitted to the light/dark test as described in the previous section.

### Statistical analysis

All analyses were performed in triplicate and data expressed as mean (n = 3) ± Standard Deviation (SD) using one-way and two-way Analysis of Variance (ANOVA) succeeded by Tukey's test for multiple comparison for data with normal distribution and significantly similar standard deviations with values of P < 0.05; P < 0.01 and P < 0.001. Statistical analyses and graphical presentation of the results were performed using GraphPad Prism software (version 6.1).

## Results

### Chemical profile by HPLC–MS-ESI and total flavonoid content

**Figure 1:**
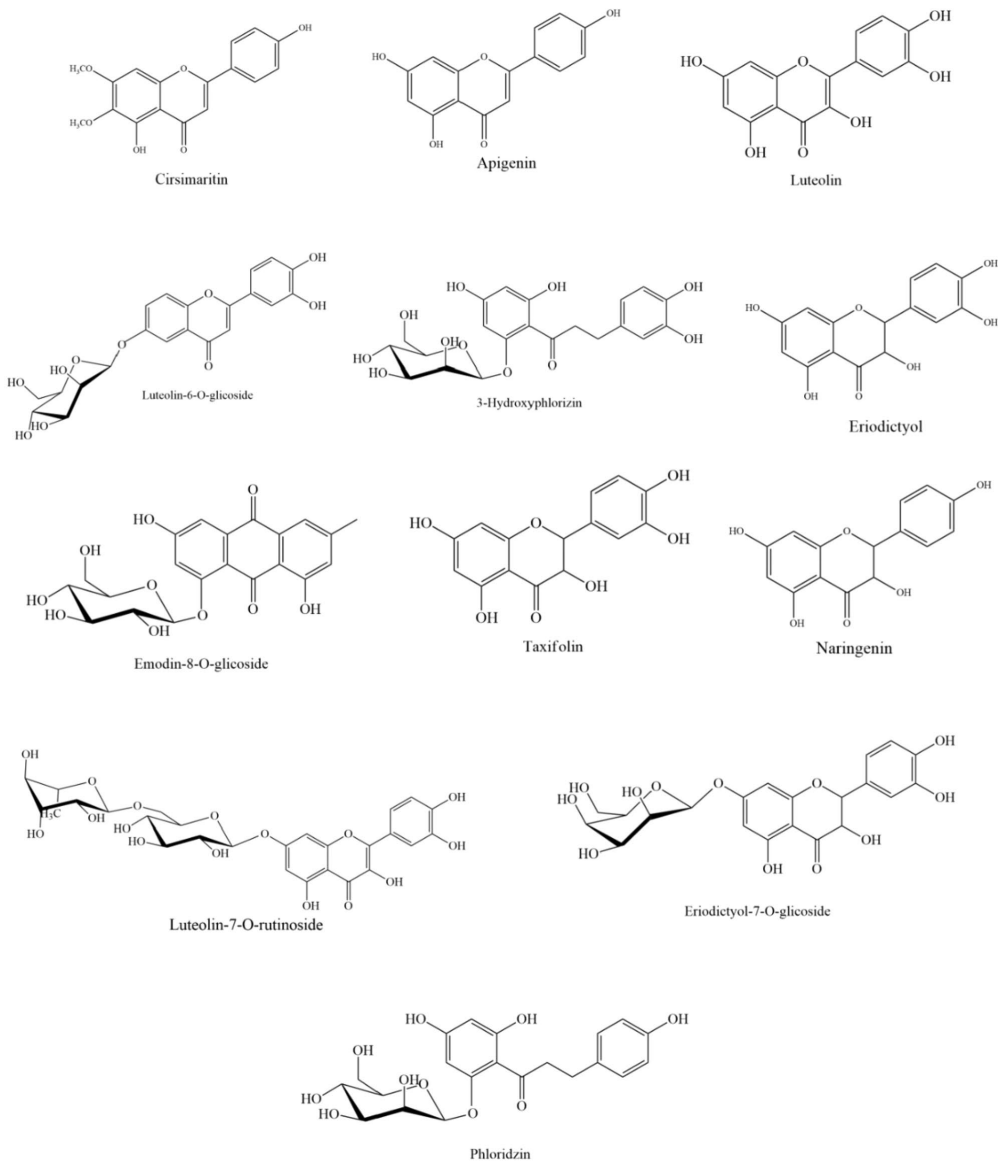
Identified compounds in the aqueous extract of *L sidoides.*

The chemical composition of *L. sidoides* extract was performed from negative mode HPLC–MS-ESI analysis from the data obtained in Table 1. 12 compounds belonging to the class of flavonoids were identified through the molecular ion mass, error (ppm), and fragmentation profile analysis of the compounds, by comparing with literature data. The chemical structures of the identified compounds are shown in Fig. 1.

**Table 1:** Negative MS fragmentation and UV–vis absorption data of the compounds detected in *L. sidoides*.

Peak no.	*t*_R_ (min.)	*m*/*z* [M − H]^−^/	Molecular formula	Error (ppm)	MS^2^/MS^3^	Tentative assignment	Refs.
1	43.2	303.0519	C_15_H_12_O_7_	1.6	MS^2^ [303.0]: 284.9; 176.9; 124.8	Taxifolin	^30^
2	43.4	449.1093	C_21_H_22_O_1_	0.5	MS^2^ [449.0]: 286.9 MS^3^ [449.0 → 286.9]: 150.9 MS^4^ [449.0 → 286.9 → 150.9]: 106.9	Eriodictyol-7-O-glicoside	^31^
3	47.2	451.1231	C_21_H_24_O_1_	− 2.6	MS^2^ [451.0]: 288.9 MS^3^ [451.0 → 288.9]: 270.9; 166.8; 124.9	3-Hydroxyphlorizin	^32,33^
4	48.5	447.0940	C_21_H_20_O_1_	− 1.3	MS^2^ [447.0]: 284.9 MS^3^ [447.0 → 284.9]: 240.9; 198. 8; 174.9; 150.8; 132.9	Luteolin-6-O-glicoside	^31^
5	51.9	435.1296	C_21_H_24_O_1_	0.1	MS^2^ [435.0]: 272.9 MS^3^ [435.0 → 272.9]: 166.8	Phloridzin	^32,34^
6	52.6	431.0994	C_21_H_20_O_1_	− 2.4	MS^2^ [431.0]: 268.9 MS^3^ [431.0 → 268.9]: 224.8	Emodin-8-O-glicoside	^35^
7	55.5	287.0596	C_15_H_12_O_6_	− 2.0	MS^2^ [287.0]: 268.8; 150.8; 124.9; 106.9	Eriodictyol	^36^
8	60.6	271.0619	C_15_H_12_O_5_	− 2.2	MS^2^ [270.9]: 176.8; 150.8; 118.9	Naringenin	^36^
9	62.3	285.0408	C_15_H_10_O_6_	− 1.3	MS^2^ [284.9]: 266.9; 256.8; 242.9; 240.9; 216.9; 198.9; 174.9; 150.9; 132.9	Luteolin	^32,36^
10	64.1	593.1489	C_27_H_30_O_1_	− 2.5	MS^2^ [593.0]: 446.9; 284.9 MS^3^ [593.0 → 284.9]: 240.8; 198.7; 174.8; 150.9; 132.9	Luteolin-7-O-rutinoside	^37–39^
11	66.9	268.0459	C_15_H_10_O_5_	− 1.5	MS^2^ [268.9]: 224.8; 226.9; 200.9; 150.9; 148.8	Apigenin	^40,41^
12	70.0	313.0735	C_17_H_14_O_6_	− 1.3	MS^2^ [313.0]: 297.9; 282.9; 268.9	Cirsimaritin	^42^

The chromatographic profile demonstrated the presence of phenolic compounds in the extract. From this, a quantification of total flavonoids was performed in order to verify the estimated amount of this class in the extract. The value found was 6.3 ± 0.8 µg eq.Q/g of the extract.

### Toxicity in *Drosophila melanogaster*

**Figure 2:**
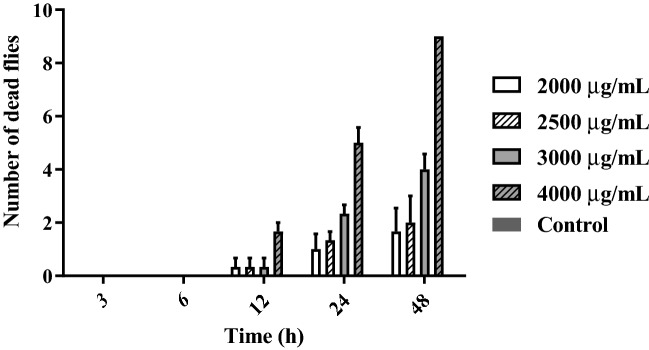
*L. sidoides* aqueous extract mortality tests against Drosophila melanogaster. The graph shows the mortality of *D. melanogaster* when exposed to concentrations of 2000, 2500, 3000, as well as 4000 μL/mL of the extract at 48 h of exposure compared to the control group.

Aqueous extract of *L. sidoides* exhibited the highest toxicity against *Drosophila melanogaster* at the concentration of 4000 μg/mL after 24 h of exposure. At concentrations of 2500 and 3000 μg/mL, an increase in the number of fly deaths could be observed after 48 h of exposure to the product. This result suggests that higher concentrations require less time of product exposure to cause mortality of *D. melanogaster* larvae. Figure 2 shows the number of deaths per concentration of the extract.

It was possible to observe that after the longest exposure time, even at the lowest concentrations, there was mortality. Thus, the results indicate a significant difference in the exposure time to the extract.

### Negative geotaxis

**Figure 3:**
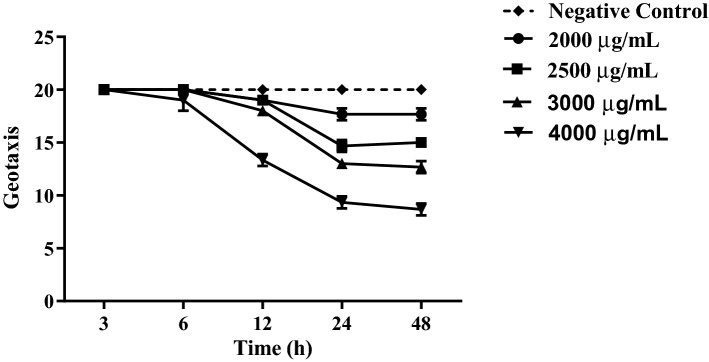
Negative geotaxis test in *Drosophila melanogaster* when exposed to different concentrations of *L. sidoides* aqueous extract in 48 h compared to the control group.

Negative geotaxis results revealed that the extract showed damage to the locomotor system of the flies at the concentration of 4000 μg/mL after 12 h of exposure. The concentrations 2000, 2500, and 3000 μg/mL differed from the control group after 24 h of exposure. These results indicate a relationship between dose and damage to the locomotor system of the flies, as shown in Fig. 3.

### Cytotoxicity on erythrocytes

**Figure 4:**
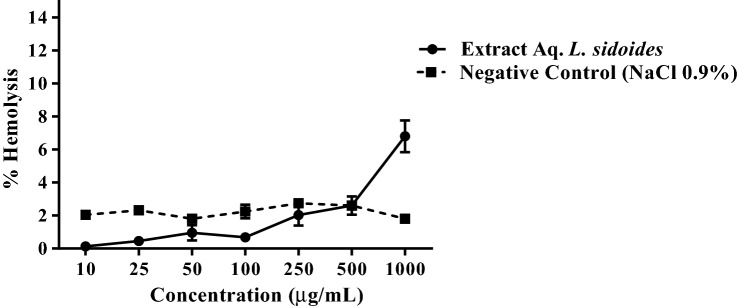
hemolytic effect on blood cells treated with aqueous extract of *L. sidoides*. The percentage of hemolysis was plotted for each concentration against the control with 100% hemolysis (NaCl 0.12%).

According to the results obtained in this study, the extract did not show cytotoxicity in blood cells, even at the highest doses (Fig. 4). When compared to the control with 0.9% saline, there was no significant difference in the different concentrations tested.

### Morphological analysis

**Figure 5:**
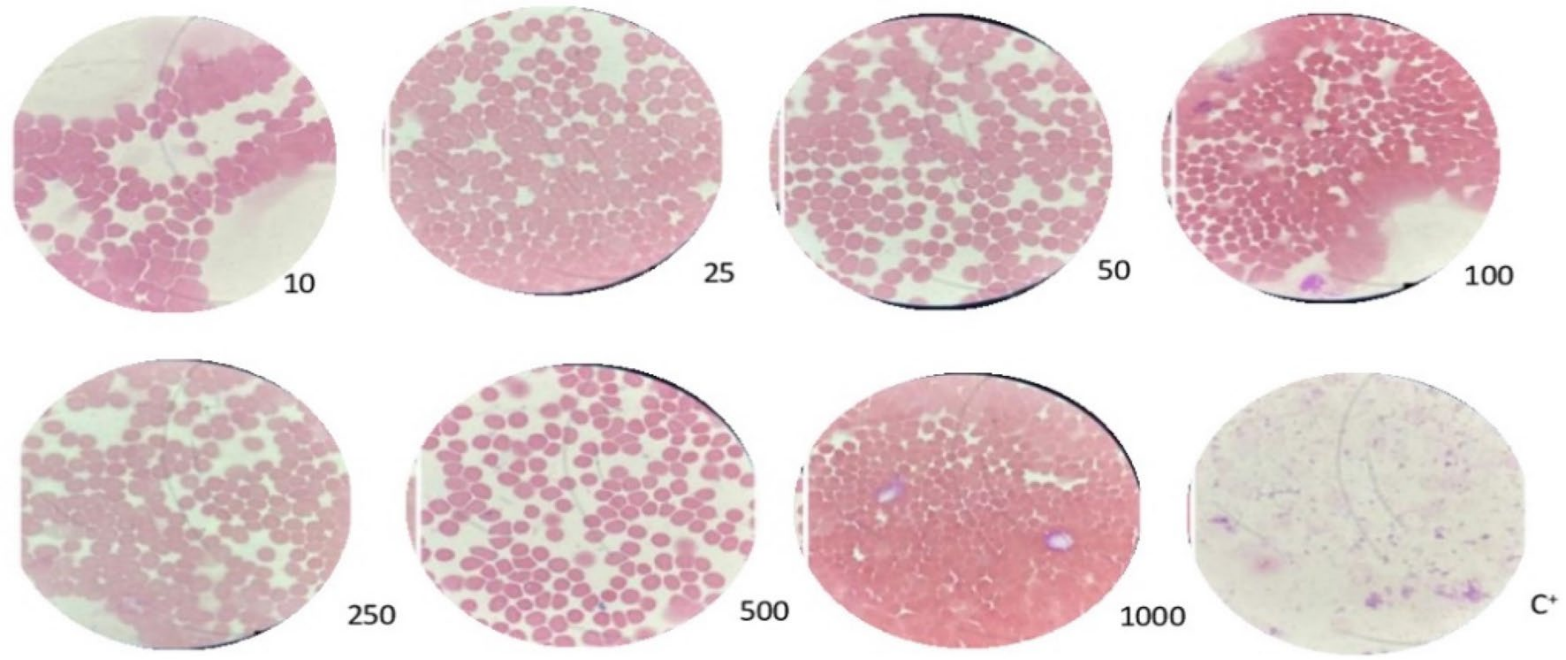
Comparison of erythrocyte morphology treated with different concentrations of *L. sidoides* aqueous extract and positive control (C^+^) with distilled water.

Comparing the morphological analysis of cells treated with *L. sidoides* extract, and the control group containing distilled water, no changes in cell structure were observed, demonstrating that different concentrations of *L. sidoides* aqueous extract do not morphologically affect blood cells (Fig. 5).

### Cytotoxic activity against *Artemia salina*

The assay against *Artemia salina* Leach revealed low toxicity at the concentrations tested, not being possible to determine the IC_50_ value.

### Zebrafish bioassays

#### Assessment of locomotor activity (Open Field Test)

**Figure 6:**
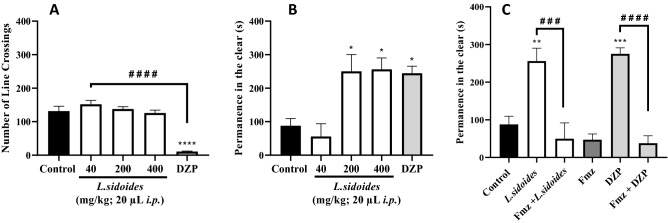
Effect of the aqueous extract of *L. sidoides* on the locomotor activity of adult zebrafish (*Danio rerio*) in the Open Field Test (0-5 min) (**A**), in the light & dark test (**B**) and on the mechanism of action anisolytic (**C**). DZP—diazepam (10 mg/kg; 20 µL; i.p.). Control—3% DMSO (20 µL; *i.p*.). Fmz—Flumazenil (4 mg/kg; 20 µL; *i.p*.). Values represent the mean ± standard error of the mean (S.E.P.M.) for 6 animals/group. ANOVA followed by Tukey (*p < 0.05, **p < 0.01, ****p < 0.0001 vs. vehicle; ^###^p < 0.001, ^####^p < 0.0001 vs DZP or *L. sidoides*).

According to the results of the open field test (Fig. 6A), it was observed that the aqueous extract of *L. sidoides* did not cause a reduction in locomotion at the doses tested, significantly different from the effect of DZP (^####^p < 0,0001 vs. DZP).

### Anxiolytic activity (light & dark test)

Higher doses of the extract increased the animals' permanence time in the clear area of the aquarium, similar to the group treated with DZP (*p < 0.05 vs. Control) (Fig. 6B), indicating an anxiolytic effect of the aqueous extract of *L. sidoides* in adult zebrafish.

### Assessment of GABAergic neuromodulation

The mechanism of anxiolytic action via GABA was determined by pretreatment with flumazenil. The highest dose of the extract with anxiolytic effect (400 mg/kg) and DZP (10 mg/kg) had the anxiolytic effect significantly blocked by flumazenil (^###^p < 0.001; ^####^p < 0.0001 vs. *L. sidoides* and DZP), returning to present anxiety behavior when remaining most of the time in the dark area of the aquarium (Fig. 6C).

In relation to the acute toxicity test, the extract was not toxic against zebrafish up to 96 h of analysis as shown in Table 2. This test allows establishing safe dose ranges and characterizing adverse effects for the use of extracts such as *L. sidoides*^43^.

**Table 2:** Acute toxicity test results (96 h).

Sample	Mortality				96 h DL_50_ (mg/kg)/IV
	CN	D1	D2	D3	
Extract *L. sidoides*	0	0	0	0	> 400

CN- Negative control group: DMSO 3%. D1—Dose 1 (40 mg/kg). D2—Dose 2 (200 mg/kg). D3—Dose 3 (400 mg/kg). LD50-Lethal dose to kill 50% of adult Zebrafish; IV-confidence interval.

## Discussion

Several studies have demonstrated the chemical composition of *Lippia* species. Twelve compounds were isolated from the ethanolic extract of the *L. sidoides*, as follows: 3-O-acetyleolic acid, methyl 3,4-dihydroxybenzoate, lapachenol, tecomaquinone, tectoquinone, tectol, acetylated tectol, quercetin, luteolin, glucoluteolin, taxifolin, isolariciresinol, and lippsidoquinone, corroborating in part with the compounds identified in this study^44^.

In phytochemical triage studies, *L. sidoides* extract exhibited secondary metabolite classes such as alkaloids, flavonoids, tannins, and terpenoids, which are associated with different biological activities^45^. Ethanolic extracts from six *Lippia* species were chemically investigated; among the compounds, naringenin, phloretin, (2S)- and (2R)-3′,4′,5,6-Tetrahydroxyflavanone-7-O-β-glucopyranoside, (2S)- and (2R)-Eriodictyol 7-O-β-d-glucopyranoside, 6-Hydroxyluteolin-7-O-β-glucoside, aromadendrin, asebogenin, and sakuranetin were present in the *L. sidoides* extract^46^. This result may be associated with the type of extract, and that aqueous extracts of these species have a higher rate of quantification of flavonoids, which is associated with the polarity of the solvent and the quantification method used^47^.

In the present study low toxicity of the aqueous extract of *L. sidoides* was demonstrated in different methods. The essential oil extracted from the leaves of this species has shown important pharmacological properties. In an analysis of the periodontal anti-inflammatory effect, it was verified that the nanostructured gel of the essential oil reduces tissue damage by decreasing the activity of the myeloperoxidase enzyme^48^. In another study, the essential oil of *L. siodides* showed anti-inflammatory activity by different models using mice, with thymol being the major representative in the chemical composition^49^. The toxicity results obtained in this study are essential to ensure the eventual use of this species in new works, especially in biological assays.

The presence of flavonoids in the chemical composition of the aqueous extract of *L. sidoides* can be associated with the low cytotoxic activity observed in this study. In concordance with this work, in Fernandes et al.^50^ evaluated the ability of the flavonoid vitexin to cause genotoxicity in two *D. melanogaster* strains, and no significant changes were observed in the DNA of the species. Another study also investigated the flavonoids kaempferol, quercetin and quercetin 3β-d-glucoside for their genotoxic action; the results showed that the flavonoids do not induce somatic mutations in flies, with the exception of quercetin at the concentration of 50 µM^51^.

In the study with *L. alba* essential oil is able to immobilize *D. melanogaster* flies after 150 min of exposure^52^. The oil effect was reversible, and no mortality of flies was observed, which is in agreement with our study. In another work it was shown that that the flavonoid hesperidin tends to decrease the deleterious effects caused to the locomotor system of flies by Fe action, as well as decreases the mortality of larvae exposed for long periods to this element^53^.

From the results obtained for cytotoxicity on erythrocytes, no serious damage to the cell membrane was observed compared to the positive control of 0.12% saline. This result suggests that there is no cytotoxicity at the concentrations tested in human erythrocytes. In contrast, the essential oil of *L. microphylla* has moderate toxicity in erythrocytes of mice with 50% hemolysis at a concentration of 300 μg/mL^54^. In an osmotic stability study with the aqueous extract of *Lippia* sp., the ability of this extract to stabilize the erythrocyte cell membrane was observed, preventing the entry of NaCl and consequently its lysis^55^. These results indicate a possible relationship between the presence of flavonoids and the preservation of erythrocyte cell membranes against oxidative damage.

The presence of compounds such as flavonoids in extracts has been proven to be an important factor in stabilizing erythrocyte membranes exposed to factors that induce cytilysis; this response is associated with the antioxidative capacity that these substances play^56^. One of the factors that alter the erythrocyte membrane making it susceptible is the degradation of proteins. Flavonoids that present hydroxyl groups at C3 tend to have a higher protein anti-degrading activity^57^, suggesting that the action of *L. sidoides* extract may be related to the presence of these compounds.

As observed in previous assays, the *L. sidoides* extract also showed low toxicity against A. salina larvae. Different results were observed in the study with the methanol/water extract of *L. multiflora* in which a high toxicity was observed, with an LC_50_ result of 1.1 µg/mL^58^. Analysis of methanolic extracts prepared from different parts (stem, leaf, and flowers) of *L. citriodora* showed that all extracts exhibited significant lethality against A. salina, with their composition being rich in tannins, polyphenols, triterpenes, catechins and alkaloids^59^.

Essential oil of *L. alba* exhibited significant toxicity against *A. salina*, showing IC_50_ of 53.01 µg/mL; the result observed may be associated with terpenic compounds present in its essential oil^60^. Samples that present IC_50_ lower than 1000 µg/mL in cytotoxic analyses are considered significant according to the study by^21^.

Locomotor activity is a behavioral analysis parameter used to evaluate chemicals acting on the central nervous system (CNS) in zebrafish^61^. From this perspective, regarding the biological effects reported for the genus *Lippia*, its properties on the central nervous system are highlighted by series of studies^62–64^.

Differently from the results obtained, *Dianthus caryophyllus* essential oil was evaluated for its toxicity in juvenile zebrafish, showing LC_50_ value of 18.18 ± 5.52 mg/L after 96 h of exposure. The sensitivity caused by this oil can be associated to its major compound eugenol^65^. On the other hand, similar results were found with *Ocimum basilicum* essential oil showed no alteration in locomotor activity or death in adult zebrafish, even after 96 h of analysis, corroborating the results obtained in this study^66^. The zebrafish has been a wellaccepted model in vitro toxicological tests performed in laboratory^67^. According to the result, the aqueous extract did not cause motor impairment or any side effects in the adult zebrafish during the 96 h of analysis, unlike DZP which, in addition to causing changes in the animals' locomotion, is known in the clinic to cause side effects and dependence^68^, for this reason the extract is promising for studies involving its effects on the CNS.

The anxiolytic effect of the extract was investigated through the light test on adult zebrafish. The treated animals showed an anxiolytic effect, as substances that fight anxiety increase the time spent by the animals in the clear region of the aquarium, while drugs that induce anxiety decrease it^28^. In the review study on the pharmacological effects of the *Lippia* genus on the central nervous system, several studies were identified demonstrating the sedative, anxiolytic, and anti-convulsant effects of plants of the genus, being responsible for these pharmacological actions nonvolatile substances such as phenylpropanoids, flavonoids and/or or inositols^69^.

Several studies on the mechanism of action of anxiolytic compounds in adult zebrafish have used flumazenil to investigate its possible effects through modulation at the binding site of benzodiazepines on the GABAa receptor^68,70,71^. In this study, tests with flumazil were performed to verify its interference in the action of the extract. Pre-treatment with flumazenil reversed the activity of the extract, as the animals returned to show an anxious behavior similar to the control, thus, probably the anxiolytic effect of *L. sidoides* extracts may occur through interaction with the binding site of benzodiazepines^69^.

The observed results show the potential of *L. sidoides* extract as an anxiolytic agent. However, there is still much to be explored about this activity, with regard to the prolongation of its effect. An alternative that can enhance the effect of this extract is its encapsulation in nanoparticles. Among the benefits of using nanoparticles is controlled delivery of the active ingrediente^72^.

In a previous study, it was verified that the preparation of a nanogel containing thymol extracted from the essential oil of *L. sidoides* promotes the reduction of periodontitis in mice^48^. This result suggests that there may be a significant improvement in pharmacological activity when the substance is encapsulated in nanoparticles. Thus, this may be a new strategy for the continuation of this study. Aiming for significant improvements in results.

## Conclusions

From the analysis of the results, it was possible to verify the presence of phenolic compounds of the flavonoid class present in the aqueous extract of *L. sidoides*; these compounds are involved in important biological activities as an antioxidant. In general, the extract did not show cytotoxic activity by the methods developed, as well as it was not toxic in adult zebrafish. The anxiolytic effect observed may be associated with GABAergic receptors. These findings suggest safety in the use of the species for the development of new studies for other biological activities, as well as for the development of specialized clinical trials, as well as for use by populations in traditional medicine.

## Supplementary Information

Supplementary Information.

## Data Availability

All data generated or analysed during this study are included in this published article (and its Supplementary Information files).
