# Supplementary material for: Analysis toxicity by different methods and anxiolytic effect of the aqueous extract Lippia sidoides Cham
Source: Sci Rep. 2022 Nov 30;12:20626. doi: 10.1038/s41598-022-23999-9 (PMC9712538; doi:10.1038/s41598-022-23999-9)
Supplement: Supplementary file 1 — Supplementary Information. [file 41598_2022_23999_MOESM1_ESM.pdf]

Drosophila melanogaster toxicity

	Concentration µg/ mL														
	2000			2500			3000			4000			control		
hours															
3	0	0	0	0	0	0	0	0	0	0	0	0	0	0	0
6	0	0	0	0	0	0	0	0	0	0	0	0	0	0	0
12	1	0	0	1	0	0	1	0	0	2	2	1	0	0	0
24	2	1	0	2	1	1	2	2	3	5	6	4	0	0	0
48	3	2	0	4	1	1	4	3	5	9	9	9	0	0	0

Geotaxis

	concentration µg/ mL														
	2000			2500			3000			4000			control		
hours															
3	20	20	20	20	20	20	20	20	20	20	20	20	20	20	20
6	20	20	20	20	20	20	20	20	20	20	19	18	20	20	20
12	19	19	19	19	19	19	18	18	18	14	13	13	20	20	20
24	18	17	18	15	14	15	13	13	13	10	9	9	20	20	20
48	18	18	17	15	15	15	13	13	12	9	9	8	20	20	20

Cytotoxicity in erythrocytes

concentration µg/ mL	extract hemolysis %			negative control		
10	0.081	0.200	0.100	2.04	2.04	2.04
25	0.530	0.440	0.400	2.32	2.32	2.32
50	1.500	0.690	0.690	1.80	1.80	1.80
100	0.610	0.850	0.570	1.80	2.61	2.32
250	2.500	1.300	2.300	2.74	2.74	2.74
500	2.100	3.200	2.500	2.61	2.61	2.61
1000	6.400	6.100	7.900	1.80	1.80	1.80

Open field test				
Control	40	200	400	DZP
127	155	168	91	15
100	161	121	120	10
200	168	128	153	17
118	95	126	140	5
134	171	145	131	7
110	161	139	120	10

Light Dark Test				
Control	40	200	400	DZP
1	19	300	294	300
85	16	300	300	208
94	25	300	300	300
91	10	300	298	271
168	17	300	88	196
88	247	1	256	192

Mechanism of anxiolytic action					
Control	***L.sidoides***	**Fmz +*L.sidoides***	Fmz	DZP	Fmz + DZP
1	294	14	1	300	27
85	300	2	97	300	5
94	300	5	25	300	0
91	298	15	20	298	2
168	88	1	65	242	77
88	256	261	75	211	117

PTZ-induced seizure

Stage I

Control	***L.sidoides***	DZP
33	30	32
32	25	30
37	15	38
26	27	50
38	29	52
40	29	55

Stage II

Control	***L.sidoides***	DZP
42	32	107
39	36	111
40	42	87
49	40	97
36	43	79
42	37	75

Stage III

Control	***L.sidoides***	DZP
47	49	139
42	51	147
46	41	155
64	46	131
72	45	113
77	44	88
